# Improving access to mental health services for homeless people in Torbay

**DOI:** 10.1192/bjo.2021.582

**Published:** 2021-06-18

**Authors:** Tom Scott-Gatty, Tom Cant

**Affiliations:** 1 Devon Partnership Trust, Livewell Southwest NHS Trust, Shekinah; 2 Devon Partnership Trust

## Abstract

**Aims:**

Devon continues to see increasing numbers of rough sleepers despite the “Everyone In” initiative and the South West region is now behind only London and the South East nationally. The interaction of homelessness and Mental Health is complex. Mental health problems and trauma contribute to people becoming homeless as well as homelessness itself causing or exacerbating existing problems, all complicated by high rates of substance use and poor physical health. Despite the desperate need in this population they often struggle to access mental health services which are not designed with their needs in mind. Their pattern of service use is primarily that of acute services when in crisis and disengagement in the community which results in high costs and poor outcomes.

**Method:**

In July 2019 an outreach service was set up consisting of a psychiatry core trainee (Dr Tom Scott-Gatty) for half a day per week supervised by the Torbay North CMHT consultant (Dr Tom Cant) to seek opportunities to engage individuals in assessment and treatment and improve outcomes in this population. The service is primarily based at the homeless hostel in Torquay (Leonard Stocks Centre) for ease of access but is flexible about where patients are seen. Patients have been seen in various locations including medical wards, prison, on the street etc. The role includes close work and liaison with other professionals such as GPs, probation, charity sector, drug and alcohol etc. and this is integral to supporting the level of complexity seen in this population. Engagement, building relationships and trust are central to serving this vulnerable and marginalised population.

**Result:**

In January 2021 feedback forms were completed by 13 patients and 18 professionals who had used the service. Feedback was overwhelmingly positive with average overall score 9/10 from both patients and professionals. All patients reported feeling comfortable using the service and that mental health services are now easier to access. All respondents would like to see the service continue. A significant number of patients and professionals identified increasing the hours offered by the service as an area for improvement.

**Conclusion:**

This service has succeeded in improving access to mental health services for homeless people in Torbay. The service is valued by both the people it serves and the professionals supporting them. Further improvement to the service could be achieved by expanding capacity. Funding has been identified from existing local authority budgets to add a CPN to the team to achieve this.

